# GRSF1 loss in THP-1 macrophages promotes senescence-associated transcription in neighboring fibroblasts

**DOI:** 10.1038/s41598-025-11385-0

**Published:** 2025-08-14

**Authors:** Younggi Lee, Seokwoo Jo, Mi-Hee Lim, Sangik Hwang, Sohyeon Jang, Kyuseok Kim, Sung-Jin Yoon, Jian Sima, M. Laura Idda, Kyoung Mi Kim, Myriam Gorospe, Chungoo Park, Ji Heon Noh

**Affiliations:** 1 Molecular Aging Biology Laboratory (MABL), Dept of Biochemistry, College of Natural Science, Chungnam National University (CNU), Daejeon, 34134 Republic of Korea; 2 School of Biological Science and Technology, Chonnam National University, Gwangju, 61186 Republic of Korea; 3 Department of Emergency Medicine, CHA University School of Medicine, Seongnam, 13497 Republic of Korea; 4 Environmental Disease Research Center, Korea Research Institute of Bioscience and Biotechnology (KRIBB), Daejeon, Republic of Korea; 5 Laboratory of Aging Neuroscience and Neuropharmacology, School of Basic Medicine and Clinical Pharmacy, China Pharmaceutical University, Nanjing, 210009 China; 6 Institute for Genetic and Biomedical Research (IRGB-CNR), Department of Biomedical Science, University of Sassari, Sassari , Italy; 7 Department of Biological Sciences, Chungnam National University, Daejeon, Republic of Korea; 8 Laboratory of Genetics and Genomics, National Institute on Aging Intramural Research Program, National Institutes of Health, Baltimore, MD USA; 9 Dept of Biochemistry, College of Natural Science, CNU 99 Daehak-ro, Yuseong-gu, Daejeon, 34134 South Korea

**Keywords:** GRSF1, THP-1 macrophages, Cell senescence, Senescence, RNA, RNA-binding proteins, Cell biology, Molecular biology

## Abstract

Immunosenescence, the age-associated decline in immune function, is accompanied by altered macrophage phenotypes and increased chronic inflammation. Here, we examined the role of the mitochondrial RNA-binding protein GRSF1 in regulating macrophage-driven inflammation and its impact on neighboring fibroblasts. We found that macrophages differentiated from GRSF1-deficient THP-1 monocytes, particularly M(IL-4 + IL-13) macrophages, displayed elevated *IL6* mRNA expression levels and TNF-α secretion, without inducing overt senescence in macrophages themselves. Conditioned media from these macrophages triggered robust senescence-associated transcriptional changes in fibroblasts, including increased expression of *IL6*, *TNF*, *DPP4*, and *IL8*, as well as elevated SA-β-gal activity. Notably, expression of NF-κB-regulated long noncoding RNAs, such as *ANRIL* and *PACER*, was also induced in fibroblasts, suggesting the engagement of an NF-κB-linked inflammatory program. These transcriptional responses were mitigated by red ginseng extract, an anti-inflammatory compound known to suppress TNF-α signaling. Collectively, our findings suggest that GRSF1 depletion in macrophages contributes to a paracrine inflammatory niche that promotes senescence-associated gene expression in surrounding cells.

**Supplementary Information:**

The online version contains supplementary material available at 10.1038/s41598-025-11385-0.

## Introduction

Biological aging involves an irreversible decline in cellular function, which in turn leads to a decrease in the resilience of organisms^1^. During this process, the immune system also exhibits a time-dependent deterioration, which is now coined immunosenescence (based on a concept proposed earlier by Walford) and is associated with the greater susceptibility to infectious and age-related disorders observed in the elderly^2,3^. Previous studies have identified several cellular events that accelerate immunosenescence, such as oxidative stress, mitochondrial dysfunction, epigenetic changes, cytokine secretion, disrupted proteostasis, and persistent DNA damage, all of which are hallmarks of senescence^4–8^. However, it is still unclear which molecules play a key role in each process^3^.

Macrophages, an essential component of the innate immunity, ultimately contribute to the activation of the adaptive immune system through the release of a variety of cytokines or direct interactions with neighboring cells^9^. During the aging process, the inflammatory profile of macrophages is altered, which serves as the senescence-associated secretory phenotypes (SASPs), contributing to tissue damage and dysfunction. Recent studies have shown that senescent macrophages, which accumulate under various stresses, are associated with immunological aging as well as systemic age-related diseases^10–13^. Senescent macrophages display molecular features commonly observed in senescent cells, including a rise in cyclin-dependent kinase inhibitors (e.g., p16INK4a, p21Cip1, etc.) and the activation of SA-β-Gal^11,14^. Such changes in senescent macrophages are observed in age-related diseases, including cancer^12,15,16^. To date, very little is known about the molecular details of senescent macrophages; however, it is clear that during their senescence, impaired phagocytic and autophagic functions are observed, along with altered metabolic signaling^17–19^. In this regard, a recent study suggested that during macrophage senescence, EP2/PGE2 signaling converts glucose to glycogen, thereby reducing glucose flux and mitochondrial respiration^20^. These findings suggest that macrophage senescence is accompanied by functional defects in molecules essential for mitochondrial maintenance.

G-rich RNA sequence binding factor 1 (GRSF1) is an RNA-binding protein that forms phase-separated ribonucleoprotein complexes (RNPs) in the mitochondrial matrix to aid organelle gene expression^21^. Previously we have shown that GRSF1 contributes to the functional fitness of mitochondria by ensuring that energy metabolism in the organelle is efficient. For example, depleting GRSF1 increases reductive stress, which is confirmed by a significant drop in the NAD^+^/NADH ratio^22^. Such redox imbalance can lead to a shortage of NAD+, which reduces the activity of NAD^+^-dependent sirtuins, one of the main causes of cell senescence^23,24^. In fact, our recent data have shown that GRSF1 levels significantly decrease during cell senescence, which induces the nuclear DNA damage via elevated mitochondria-sourced reactive oxygen species^25^. Meanwhile, a recent report has shown that innate immune challenges disrupt mitochondrial NAD^+^ synthesis and over-activate the inflammatory phenotypes of macrophages, leading to host immune imbalance, which is consistent with features seen in aged macrophages^26^. Interestingly, in addition to the aforementioned effects on availability of NAD^+^, depleting GRSF1 significantly increases IL6 secretion from LPS-challenged monocytes, which in turn promotes the senescence of neighboring fibroblasts^25^.

Here, we investigated whether GRSF1 deficiency alters macrophage differentiation and inflammatory profiles, subsequently promoting senescence-associated gene expression in neighboring fibroblasts. Our findings reveal a critical role of GRSF1 in modulating macrophage-driven inflammation and suggest its involvement in the regulation of cellular senescence and age-related inflammatory conditions.

## Methods

### Cell culture and reagents

THP-1 monocytes were cultured in RPMI-1640 (Biowest, L0498) containing 10% heat-inactivated fetal bovine serum (FBS, Gibco, 16000044), antibiotics, and antimycotics (Gibco, 15240-062). Generating GRSF1-deficient THP-1 monocytes has been described previously^25^. WI-38 human diploid fibroblasts were purchased from the Coriell Cell Repositories and cultured in DMEM (Corning, 10-013-CVRC) supplemented with 10% FBS, antibiotics, antimycotics, and non-essential amino acids (Gibco, 11140050). WI-38 fibroblasts were used at population doubling levels (PDLs) between 36 and 38. Red ginseng extracts were obtained from the Korea Tobacco and Ginseng Cooperation (Daejeon, Korea).

### Differentiation and polarization of M(LPS + IFN- γ) or M(IL-4 + IL-13) macrophages

To obtain polarized macrophages (Mφ), THP-1 cells expressing shCTRL or shGRSF1 were differentiated into naïve (Mφ by treatment with 50 ng/mL of phorbol 12-myristate 13-acetate (PMA) for 48 h. naïve Mφ were further cultured for another 48 h in the presence of a combination of LPS (100 ng/mL) + IFN-γ (20 ng/mL) or IL4 (20 ng/mL) + IL13 (20 ng/mL) to polarize them into classically activated or alternatively activated Mφ, respectively. We named naïve macrophages as M(-), classically activated macrophages as M(LPS + IFN-γ), and alternatively activated macrophages as M(IL4 + IL-13)^27^. PMA (P1585) and LPS (L2762) were from Sigma Aldrich. IFN-γ (#300-02), IL4 (#200-04), and IL13 (#200-13) were from Peprotech.

### Collection of conditioned medium and co-culture

M(LPS + IFN- γ) or M(IL-4 + IL-13) macrophages derived from control or GRSF1-deficient THP-1 monocytes were incubated for 24 h with conditioned medium (CM) consisting of RPMI 1640, 1% antibiotics/antimycotics, and 0.1% bovine serum albumin (BSA, Mpbio, 0216006980). After the culture period, the medium was collected and centrifuged at 2000 rpm for 5 min and the supernatant was used in further applications. The supernatant derived from M(LPS + IFN- γ) macrophages was named M(LPS + IFN- γ)CM and the supernatant derived from M(IL-4 + IL-13) macrophages was named M(IL-4 + IL-13)CM.

### RNA isolation, reverse transcription, and quantitative real-time PCR

Total RNA was extracted in Tri reagent (Invitrogen, AM9738) using Quick RNA Miniprep kit (Zymo research, R2052) following the manufacturer’s protocol. cDNA was synthesized using random hexamers (Thermo Scientific, SO142) and reverse transcriptase (Thermo Scientific, EP0442). Reactions for qPCR amplification contained SYBR Green master mix (Kapa Biosystems, KK4605) and were performed using an Applied Biosystems 7300 instrument. A list of primers is provided in the Supplementary Table 1.

### Western blot analysis

Cells were lysed in RIPA buffer supplemented with 1 mM PMSF and a protease and phosphatase inhibitor cocktail (Thermo Scientific, 1861282). Whole-cell lysates (15 µg of protein) were separated by SDS-polyacrylamide gel electrophoresis and transferred to nitrocellulose membranes. The membranes were hybridized with antibodies recognizing specific target proteins, and the protein bands were visualized by enhanced chemiluminescence (Kindle Biosciences) using a KwikQuant Imager (Kindle Biosciences, LLC, D1001). Primary antibodies were used that recognized p21 (05-345, Sigma Aldrich), GRSF1 (703097, Invitrogen), DPP4 (ab129060, Abcam), p16 (551154, BD Bioscience), GAPDH (SC-32233, Santa Cruz), ACTB (β-actin; SC-47779, Santa Cruz), and phsopho-p65 (#3033, Cell signaling). HRP-conjugated secondary antibodies were from Kindle Biosciences.

### Senescence-Associated β-Galactosidase (SA-β-gal) Activity

WI-38 fibroblasts were cultured for 24 h with conditioned medium (CM) collected from GRSF1-deficient M(IL-4 + IL-13) macrophages. Subsequently, cells were stained to assess senescence-associated β-galactosidase (SA-β-gal) activity according to the manufacturer’s instruction (Cell Signaling Technology). The percentage of senescent cells was determined by calculating the ratio of SA-β-gal-positive cells to the total cell count (*n* = 3).

### Cytokine array

Cells differentiated from THP-1 monocytes to M(IL-4 + IL-13) macrophages were cultured in conditioned medium for 24 h, after which the medium was harvested for cytokine array. Human Cytokine Array 5 Maps (RayBiotech, AAH-CYT-5) were used to determine the relative quantities of 80 cytokines following the manufacturer’s instructions.

### Cell proliferation

The viability of WI-38 cells was measured by 3-(4,5-dimethylthiazol-2-yl)−5-(3-carboxymethoxyphenyl)−2-(4-sulfophenyl)−2 H-tetrazolium (MTS) reagent (Promega, G3580). Briefly, cells were seeded in 96-well plates and exposed to different concentrations (0–1000 µg/mL) of red ginseng extract in the presence or absence of serum for 24 h. After incubation for 24 h, 20 µL of MTS reagent was added to each well, and cells were further incubated for 3 h. Absorbance at 490 nm was determined by using a microplate reader.

### Statistical analysis

All statistical analyses were performed using GraphPad Prism 10. Comparisons between two groups were conducted using unpaired Student’s t-test. For experiments involving multiple groups, statistical significance was determined using one-way analysis of variance (ANOVA) followed by Tukey’s multiple comparison test. All data are presented as mean ± SD, and a p-value < 0.05 was considered statistically significant.

## Results

### Depleting GRSF1 induces monocyte senescence and pro-inflammatory polarization of M(IL-4 + IL-13) macrophages

**Fig. 1:**
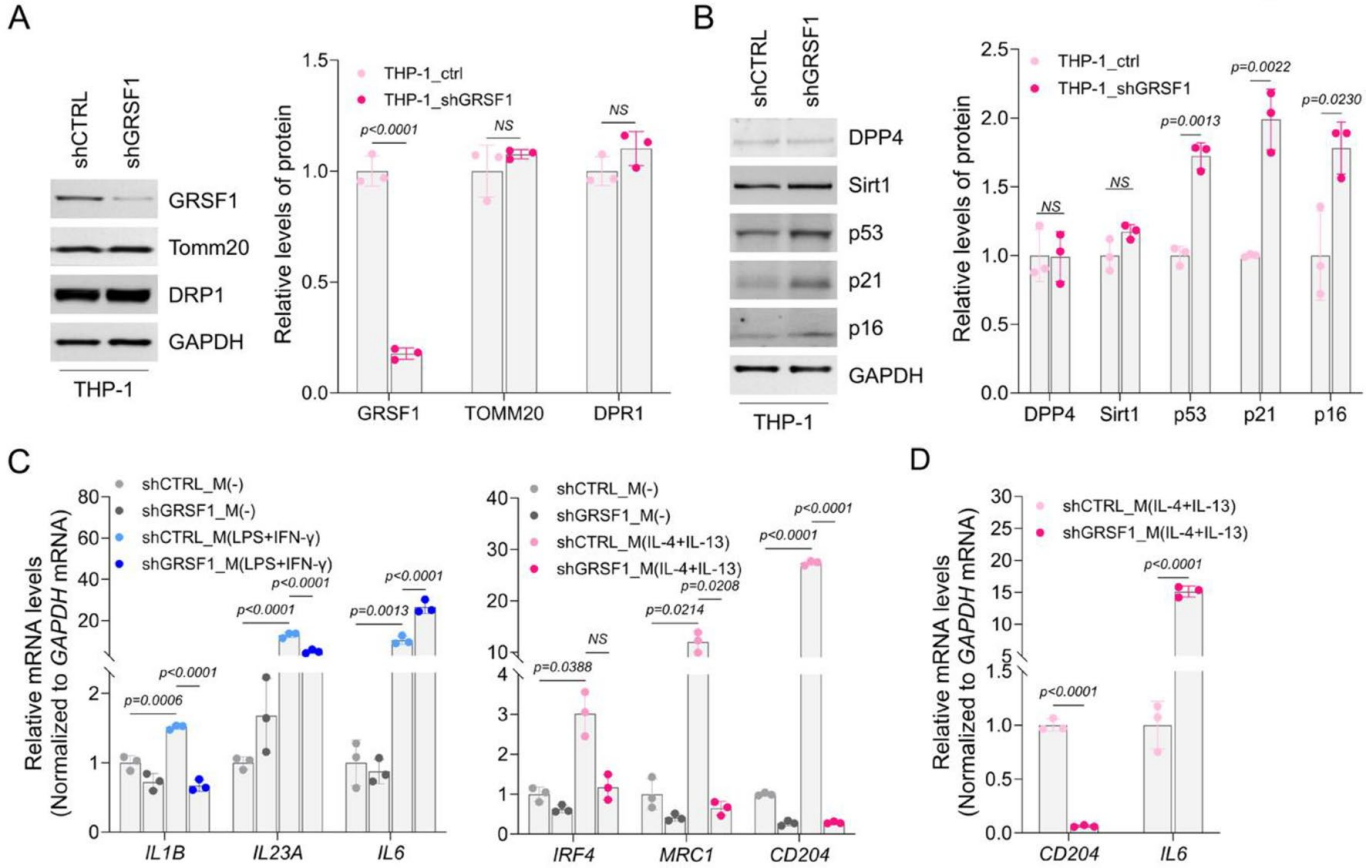
Depleting GRSF1 interferes with macrophage polarization. (**A**) THP-1 monocytes were transduced with lentiviruses expressing shGRSF1 or shCTRL. Expression levels of GRSF1 and loading controls (TOMM20, DRP1, and GAPDH) were analyzed by Western blot (*left*). Band intensities were quantified and presented as a bar graph (*right*). (**B**) The expression levels of senescence markers (DPP4, Sirt1, p53, p21, and p16) and loading control (GAPDH) were analyzed by Western blot (*left*). Band intensities were quantified and presented as a bar graph (*right*). (**C**) Total RNA was isolated from M(-), M(LPS+IFN-γ), and M(IL-4+IL-13) macrophages differentiated from THP-1 monocytes expressing shGRSF1 or shCTRL. The mRNA levels of M(LPS+IFN-γ)-specific (*IL1B*, *IL23A*, and *IL6*) and M(IL-4+IL-13)-specific markers (*IRF4*, *MRC1*, and *CD204*) were determined by RT-qPCR analysis. Relative mRNA expression was normalized to the M(-) group and presented as bar graphs. (**D**) After isolating total RNA from each of shGRSF1 and shCTRL M(IL-4+IL-13) macrophages, the steady-state levels of *CD204* and *IL6* mRNAs were quantified by RT-qPCR analysis. The data in (A, B, D) represent the means ± SD of three independent experiments, and statistical significance was analyzed using Student’s t-test. In (**C**), statistical significance among four groups was assessed by one-way ANOVA, followed by Tukey’s multiple comparison test.

We previously reported increased IL6 secretion upon GRSF1 depletion in THP-1 monocytes, particularly following lipopolysaccharide (LPS) stimulation^25^. Based on these observations, we hypothesized that GRSF1 might play a role in monocyte activation and their differentiation into pro-inflammatory macrophages. To test this hypothesis, we established THP-1 cell lines stably expressing either small hairpin RNAs targeting GRSF1 (shGRSF1) or a control hairpin (shCTRL), confirming effective knockdown by Western blot (Fig. 1A; Supplementary Fig S4).

GRSF1 depletion in THP-1 monocytes led to significantly increased levels of the senescence-associated cell-cycle regulators p53, p21, and p16, while DPP4 and Sirt1 proteins remained unchanged (Fig. 1B; Supplementary Fig S4). In contrast, phosphorylation of NF-κB p65 (Ser536), a critical indicator of NF-κB pathway activation, was not significantly altered by GRSF1 knockdown (Supplementary Fig S1A; Supplementary Fig S5). Apoptosis-related proteins showed modest changes, including increased Bcl-x isoforms (Bcl-xS/L) without substantial alterations in other Bcl-2 family proteins such as Bcl-2, BID, and Bak (Supplementary Fig S1B; Supplementary Fig S5). Additionally, RT-qPCR analysis revealed no significant changes in the steady-state levels of senescence-related mRNAs including *CDKN1A*, *TP53*, *CDKN2A*, *DPP4*, and *IL6* in GRSF1-depleted THP-1 monocytes compared with control cells (Supplementary Fig S1C). Collectively, these data suggest that GRSF1 depletion primarily affects senescence-related pathways rather than directly modulating apoptosis or NF-κB-mediated inflammatory signaling in monocytes.

Based on these results, we next examined whether GRSF1 depletion affects monocyte differentiation into macrophages and subsequent inflammatory phenotypes. THP-1 monocytes expressing shGRSF1 or shCTRL were differentiated into macrophage subtypes− M(-), M(LPS + IFN-γ), and M(IL-4 + IL-13)−following established protocols (Supplementary Fig S1D). Morphological analyses revealed that M(-) macrophages displayed small, round, and adherent; M(LPS + IFN-γ) macrophages exhibited elongated spindle-like shapes; and M(IL-4 + IL-13) macrophages were rounded, flat, and displayed distinct filopodia, consistent with established phenotypes^28^. GRSF1 depletion substantially altered these morphological characteristics, markedly reducing cell elongation in M(LPS + IFN-γ) macrophages and decreasing filopodia formation in M(IL-4 + IL-13) macrophages, as quantified by morphological scoring (Supplementary Fig S1E).

We next assessed macrophage subtype-specific marker gene expression by RT-qPCR. Genes characteristics of M(LPS + IFN-γ) (*IL1B*, *IL23A*, *IL6*) and M(IL-4 + IL-13) macrophages (*IRF4*, *MRC1*, *CD204*) were induced upon differentiation compared to M(-) macrophages (Fig. 1C; shCTRL_M(LPS + IFN-γ) vs. shCTRL_M(-)). Although GRSF1 depletion generally reduced marker induction, *IL6* mRNA expression unexpectedly increased in GRSF1-deficient M(LPS + IFN-γ) macrophages, contrasting sharply with other markers (Fig. 1C; shGRSF1_M(LPS + IFN-γ) vs. shCTRL_M(LPS + IFN-γ)). Given this observation, we examined *IL6* expression in GRSF1-deficient M(IL-4 + IL-13) macrophages, detecting a pronounced increase alongside a marked decrease in the subtype marker *CD204* (Fig. 1D). Since elevated *IL6* expression is commonly associated with cellular senescence, we further assessed senescence-associated protein markers, identifying a significant increase in DPP4, a senescence-related protease involved in inflammation and immune modulation^29,30^, in GRSF1-deficient M(IL-4 + IL-13) macrophages (Supplementary Figs S1F and S5).

### GRSF1-deficient M(IL-4 + IL-13) macrophages promotes inflammatory transcriptomic responses associated with senescence in fibroblasts

**Fig. 2:**
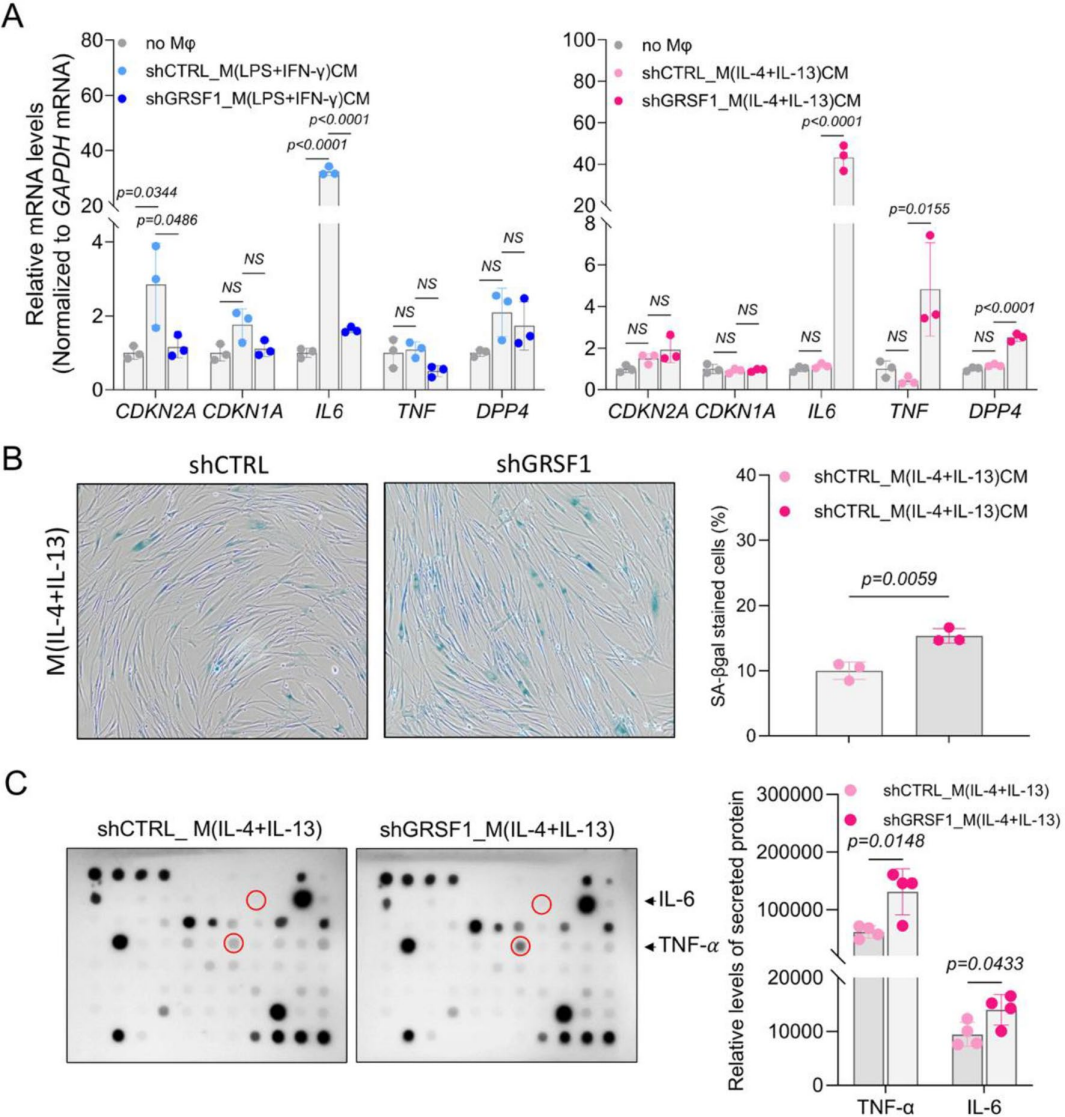
GRSF1-deficient macrophages induce the senescence in neighboring fibroblasts.(**A**) Conditioned media (CM) from M(LPS+IFN-γ) (*left*) and M(IL-4+IL-13) (*right*) macrophages expressing shGRSF1 or shCTRL were collected and used to culture pre-senescent WI-38 fibroblasts for 24 h. Serum-free medium containing 0.1% BSA (No Mφ) served as a negative control. Total RNA was isolated from recipient WI-38 fibroblasts, and the mRNA levels of cell cycle inhibitors (*CDKN2A* and *CDKN1A*) and senescence markers (*IL6*, *TNF*, and *DPP4*) were measured by RT-qPCR analysis. (**B**) SA-β-gal activity was analyzed in recipient WI-38 fibroblasts treated with CM from shCTRL of shGRSF1-expressing M(IL-4+IL-13) macrophages (*left*). SA-β-gal-positive cells were quantified and presented as a bar graph (*right*). (**C**) Conditioned media (CM) from GRSF1-deficient or control M(IL-4+IL-13) macrophages were collected, analyzed by antibody array (*left*), and quantified for released protein levels (*right*). The data in (**A**-**C**) represent the means ± SD from three independent experiments. Statistical significance in (**A**) was analyzed by one-way ANOVA followed by Tukey’s multiple comparison test, and in (**B** and **C**) by Student’s t-test.

Previous studies have highlighted the pro-inflammatory role of IL6 in cellular senescence^31,32^. As described above, *IL6* mRNA levels increased upon GRSF1 depletion in both M(LPS + IFN-γ) and M(IL-4 + IL-13) macrophages, albeit to varying degrees. To determine whether GRSF1-deficient macrophages with elevated *IL6* expression could influence neighboring cells, we cultured pre-senescent WI-38 fibroblasts for 24 h in conditioned media (CM) derived from control (shCTRL) or GRSF1-depleted (shGRSF1) macrophages (Supplementary Fig S2A). Although recipient fibroblasts displayed no evident morphological changes (Supplementary Fig S2B), significant transcriptional responses were detected by RT-qPCR analysis. Specifically, fibroblasts exposed to CM from control M(LPS + IFN-γ) macrophages exhibited increased *CDKN2A* and *IL6* mRNA expression compared to those cultured with macrophage-free control medium; notably, this induction was largely attenuated when macrophages lacked GRSF1 (Fig. 2A, left panel). Fibroblasts cultured with CM from control M(IL-4 + IL-13) macrophages showed no significant induction of any tested marker genes. In contrast, fibroblasts exposed to CM from GRSF1-deficient M(IL-4 + IL-13) macrophages displayed marked increases in *IL6*, *TNF*, and *DPP4* mRNA (Fig. 2A, right panel).

To clarify the mechanism underlying the increased steady-state levels of *IL6*, *TNF*, and *DPP4* mRNA in fibroblasts exposed to GRSF1-deficient M(IL-4 + IL-13) CM, we assessed mRNA stability using actinomycin D treatment to inhibit *de novo* transcription. No significant differences in transcript stability were observed between fibroblasts cultured with CM from control and GRSF1-deficient M(IL-4 + IL-13) macrophages (Supplementary Figs S2C and S7), suggesting that the increased mRNA abundance likely results from enhanced transcription rather than altered post-transcriptional regulation.

We next investigated whether these transcriptomic changes correlated with induction of cellular senescence in recipient fibroblasts by measuring senescence-associated β-galactosidase (SA-β-gal) activity. Fibroblasts cultured with CM from GRSF1-deficient M(IL-4 + IL-13) macrophages exhibited significantly higher SA-β-gal positivity compared to controls, clearly indicating cellular senescence induction (Fig. 2B; Supplementary Fig S6).

We hypothesized that pro-inflammatory cytokines secreted by GRSF1-deficient M(IL-4 + IL-13) macrophages might drive this senescence-associated inflammatory transcriptomic response in neighboring fibroblasts. To directly investigate this possibility, we performed cytokine array analysis to identify factors released by these macrophages. Among the cytokines detected, TNF-α exhibited the most pronounced elevation in conditioned media from GRSF1-deficient M(IL-4 + IL-13) macrophages compared to controls (Fig. 2C; Supplementary Figs S2D and S7), suggesting its potential role in mediating inflammatory senescence in recipient fibroblasts.

### GRSF1-deficient macrophages induce NF-κB-associated inflammatory gene expression in fibroblasts

**Fig. 3:**
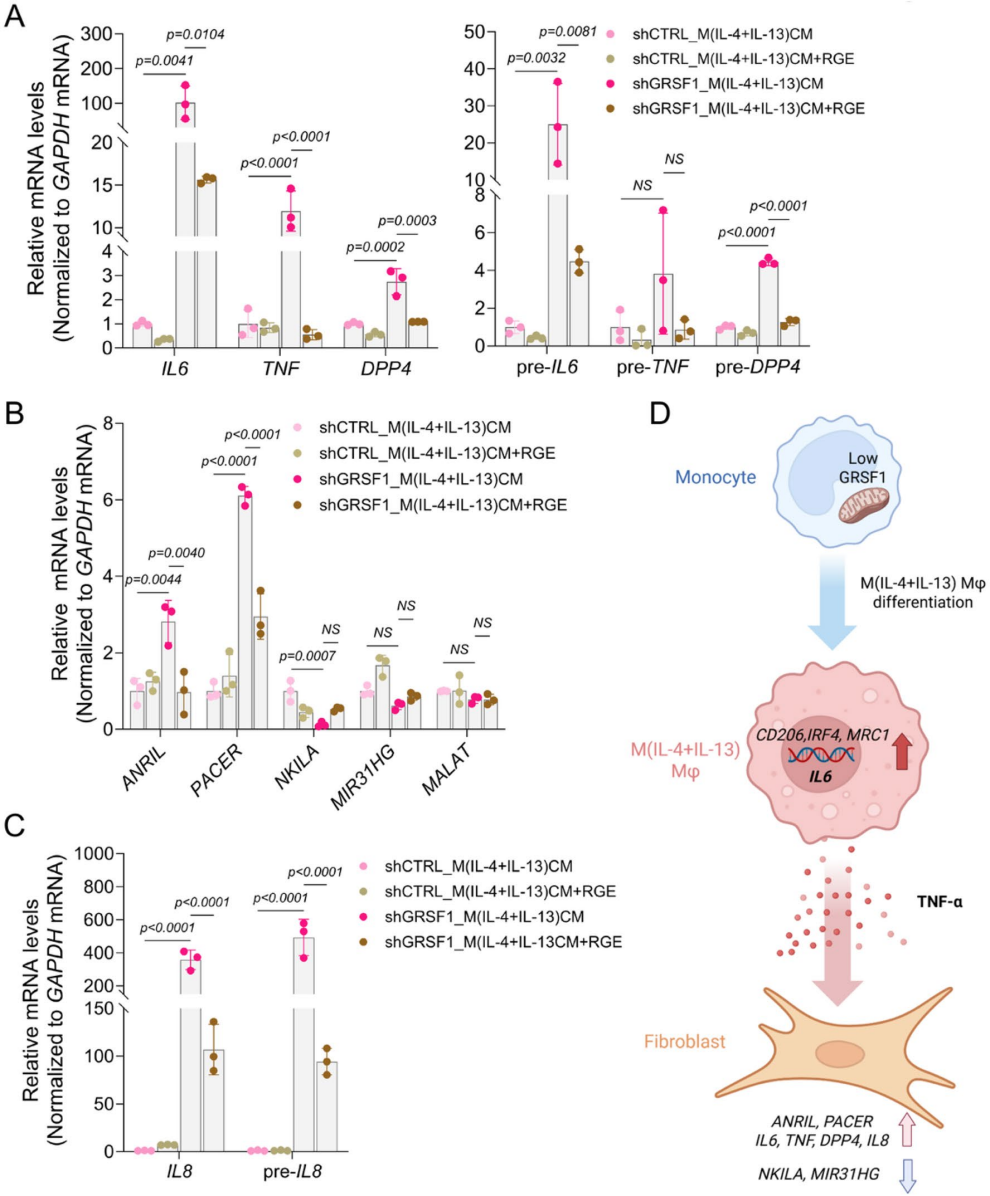
The altered inflammatory environment modulates NF-κB-dependent transcription. (**A**) WI-38 fibroblasts were cultured in conditioned media (CM) from GRSF1-deficient (shGRSF1) or control (shCTRL) M(IL-4 + IL-13) macrophages for 24 h, with or without pre-treatment with 800 µg/mL of red ginseng rextract (RGE) for 30 min. Total RNA was isolated from WI-38 fibroblasts, and both mRNA (*left*) and pre-mRNA (*right*) levels of *IL6*, *TNF*, and *DPP4* were measured by RT-qPCR analysis. (**B**) The levels of NF-κB-related lncRNAs (*ANRIL*, *PACER*, *NKILA*, *MIR31HG*, and *MALAT*) were determined by RT-qPCR analysis. (**C**) Both mRNA and pre-mRNA levels of *IL8* were measured by RT-qPCR analysis. (**D**) Proposed model: Increased *IL6* expression in GRSF1-deficient M(IL-4 + IL-13) macrophages promotes an inflammatory environment involving TNF-α. WI-38 fibroblasts exposed to these inflammatory conditions exhibit enhanced transcription of senescence-associated genes and altered expression of NF-κB-dependent transcripts. Figure created with BioRender.com. All the data in (**A**-**C**) represent the means ± SD from three independent experiments. Statistical significance was determined by one-way ANOVA followed by Tukey’s multiple comparison test.

To test the involvement of TNF-α in the transcriptomic responses of recipient fibroblasts, we used red ginseng extract (RGE), a natural compound known to suppress TNF-α signaling and inflammation. Initially, we determined the optimal RGE concentration (800 µg/mL) based on minimal cytotoxicity in cell viability assays (Supplementary Fig S3A). Subsequently, WI-38 fibroblasts were pretreated with RGE for 30 min and then exposed to CM derived from control or GRSF1-deficient M(IL-4 + IL-13) macrophages for 24 h (Supplementary Fig S3B). Consistent with the findings in Fig. 2A, CM from GRSF1-deficient macrophages again triggered robust expression of *IL6*, *TNF*, and *DPP4* mRNA in recipient fibroblasts, which was significantly attenuated by RGE pretreatment (Fig. 3A, left panel). The corresponding precursor transcripts (pre-mRNAs) were also elevated and similarly suppressed by RGE, indicating transcriptional activation via TNF-α-driven signaling (Fig. 3A, right panel).

Given the link between TNF-α and NF-κB signaling, we next examined a set of long noncoding RNAs (lncRNAs) known to be regulated by NF-κB (see Discussion section). Among them, *ANRIL* and *PACER* were significantly induced by GRSF1-deficient M(IL-4 + IL-13) CM and reduced by RGE pretreatment, whereas *MIR31HG*, *NKILA*, and *MALAT1* levels remained unchanged (Fig. 3B). While these lncRNAs have not been directly linked to senescence, the regulation of *ANRIL* and *PACER*−both known NF-κB-responsive transcripts−suggests a potential role in mediating downstream inflammatory gene expression.

Supporting this notion, *IL8* mRNA−a known downstream target of *ANRIL*−was also elevated in fibroblasts exposed to shGRSF1_M(IL-4 + IL-13) CM and suppressed by RGE pretreatment, along with increased expression of pre-*IL8* (Fig. 3C). These findings reinforce the involvement of the TNF-α-NF-κB axis in the transcriptional reprogramming observed in recipient fibroblasts. A schematic model summarizing our key findings, including how GRSF1-deficient macrophages promote inflammatory signaling and transcriptional changes in fibroblasts, is presented in Fig. 3D.

## Discussion

In this study, we investigated how GRSF1, a mitochondrial RNA-binding protein whose expression declines during senescence^25,33^, regulates macrophage inflammatory phenotypes and their paracrine effects on neighboring fibroblasts. GRSE1-deficient THP-1 monocytes differentiated into macrophages that, particularly under IL-4 and IL-13 stimulation, adopted a pro-inflammatory phenotype characterized by increased *IL6* expression and robust secretion of TNF-α. This alteration in the macrophage secretory profile is notable, as TNF-α, is a central component of the senescence-associated secretory phenotype (SASP) and a known driver of tissue inflammation and dysfunction during aging^34^.

Conditioned medium from GRSF1-deficient macrophages induced senescence-associated gene expression in pre-senescent fibroblasts, as evidenced by increased *IL6*, *TNF*, and *DPP4* mRNA levels, along with elevated SA-β-gal activity. Despite the short-term exposure, classical markers such as *CDKN2A* were not markedly induced, whereas early senescence-associated transcripts and their corresponding precursor mRNAs were robustly elevated, indicating transcriptional activation. Among the macrophage-secreted proteins, TNF-α was the most prominently elevated in GRSF1-deficient M(IL-4 + IL-13) macrophages, suggesting a potential paracrine role in triggering inflammatory gene expression in recipient fibroblasts. Although a direct causal relationship is not yet confirmed, the consistent elevation of TNF-α supports its involvement in the transcriptomic remodeling of recipient fibroblasts.

Macrophages are commonly classified as classically activated (M1) or alternatively activated (M2), although in vivo they span a functional continuum rather than distinct subtypes. Dysregulation in the M1/M2 balance is a hallmark of immunological aging^35–37^. For example, a higher M2/M1 ratio has been associated with systemic immunosuppression in aged mice^38^, whereas sustained M1-like activation contributes to chronic inflammation and tissue damage^39–41^. Among M2 subtypes, M2a macrophages are typically induced by IL-4 and IL-13^42^. In this study, we used THP-1-derived naïve macrophages polarized with IL-4 and IL-13 to generate M(IL-4 + IL-13) cells. Interestingly, GRSF1-deficient M(IL-4 + IL-13) macrophages acquired features commonly associated with M1-like cells, including elevated TNF-α secretion and substantially increased *IL6* mRNA levels. These observations suggest that GRSF1 loss reprograms M2a-polarized macrophages toward a pro-inflammatory state. Although our model relies on acute genetic depletion of GRSF1, emerging evidence raises the possibility that its expression may also decline with age in vivo. Preliminary data from natural aging mouse model suggest an age-associated reductions in *Grsf1* expression in monocyte^43^, and our analysis of publicly available human transcriptome datasets revealed a similar trend in peripheral blood monocytes (data not shown). Although it remains unclear whether GRSF1 expression naturally declines in tissue-resident macrophages during aging, our data raise the possibility that mitochondrial perturbations, potentially resulting from GRSF1 deficiency, may contribute to altered macrophage polarization and inflammatory output.

To further understand the potential regulatory mechanisms downstream of TNF-α, we examined long noncoding RNAs (lncRNAs) known to interact with the NF-κB signaling pathway. NF-κB plays a central role in inflammatory responses triggered by DNA damage and metabolic stress, both of which contribute to cellular senescence and aging^44^. Recent studies have shown that several lncRNAs modulate NF-κB activity, either enhancing or suppressing its transcriptional output^45^. Among lncRNAs that positively regulate NF-κB signaling, *ANRIL* is a well-established NF-κB-dependent transcript that promotes the transcription of inflammatory genes such as *IL6* and *IL8* by physically interacting with the transcription factor YY1^46^. *PACER* activates NF-κB signaling by sequestering the p50 subunit, allowing the p50-p65 heterodimer to accumulate at the *COX2* promoter and initiate transcription^47^. In addition, *MIR31HG* enhances NF-κB activity by promoting IκBα phosphorylation in the cytoplasm, which facilitates its own transcription in a positive feedback loop^48^. In contrast, several lncRNAs have been reported to negatively regulate NF-κB signaling. For example, *MALAT1* suppresses the expression of NF-κB-responsive genes such as *TNF* and *IL6* by binding to the p50-p65 complex and preventing its DNA interaction^49,50^. *NKILA* interferes with NF-κB nuclear translocation by binding to p65 and forming a stable *NKILA*-NF-κB-IκBα complex in the cytoplasm, thereby preventing IκB phosphorylation and limiting pathway activation^51^. In our study, fibroblasts exposed to GRSF1-deficient M(IL-4 + IL-13) CM exhibited increased expression of the NF-κB-enhancing lncRNAs *ANRIL* and *PACER*, whereas *NKILA* level as markedly suppressed. Given that *IL8* is a known downstream transcript of *ANRIL*, this finding further supports the involvement of *ANRIL*-mediated inflammatory signaling in recipient fibroblasts. In contrast, transcript levels of *MIR31HG* and *MALAT1* remained largely unchanged, although we cannot exclude the possibility of post-transcriptional regulation, including alterations in subcellular localization.

Given the potential involvement of the TNF-α-NF-κB-lncRNA axis, we investigated whether red ginseng extract (RGE), a known TNF-α suppressor, could attenuate this signaling pathway. RGE pretreatment of fibroblasts effectively reduced the expression of senescence-associated genes as well as the NF-κB–responsive lncRNAs *ANRIL* and *PACER*, supporting the involvement of this pathway in mediating the observed transcriptional response. These findings align with previous reports showing that RGE can modulate macrophage-driven inflammation in vitro and in vivo^52–55^, and further highlight its potential as a candidate anti-senescence agent. Our findings indicate that RGE can counteract the transcriptional activation induced in fibroblasts by pro-inflammatory macrophage signals. While the pharmacological efficacy of RGE remains to be validated in clinical contexts, our macrophage-fibroblast model may serve as a useful platform for screening natural compounds targeting senescence-associated inflammatory signaling.

The effects of IL6 in inflammatory and metabolic settings are known to vary depending on context. While several studies have reported anti-inflammatory effects of IL6 in adipose tissue macrophages or during endotoxemia^56^, and broader cellular contexts^57–59^, our findings suggest that IL6 upregulation in GRSF1-deficient macrophages contributes to a pro-inflammatory phenotype associated with senescence. These observations raise the possibility that maintaining adequate GRSF1 expression may help restrain low-grade systemic inflammation by limiting aberrant IL6 production. In addition, recent studies have shown that senescent macrophages exhibit impaired mitochondrial quality control, including reduced autophagy and mitophagy activity^60^. Our unpublished data indicate that GRSF1 depletion significantly impairs mitophagy, leading to the accumulation of damaged mitochondria (data not shown). This mitochondrial dysfunction may be further exacerbated by metabolic reprogramming; senescent macrophages tend to shift toward glycolysis, reflecting impaired oxidative phosphorylation capacity. Notably, such glycolytic reprogramming is also a feature of GRSF1-deficient cells^22,61^. Taken together, these findings suggest that GRSF1 loss may impact both mitochondrial turnover and energy metabolism in macrophages, although this remains to be explored in future studies.

As illustrated in the schematic model (Fig. 3D), we propose that GRSF1 deficiency drives macrophages toward a pro-inflammatory phenotype characterized by elevated TNF-α secretion. This paracrine signal activates NF-κB–driven transcriptional programs in recipient fibroblasts, leading to the induction of senescence-associated transcripts. In conclusion, our study highlights the role of GRSF1 in maintaining macrophage homeostasis and preventing the formation of a senescence-inducing microenvironment. Future work should investigate whether additional mitochondrial RNA regulators contribute to macrophage function and inflammatory polarization, and how other immune cell types such as T cells interact with dysfunctional macrophages to shape tissue-level aging responses.

## Electronic supplementary material

Below is the link to the electronic supplementary material.

Supplementary Material 1

## Data Availability

The data analyzed during the current study are available from the corresponding author on reasonable request.
