# Supplementary material for: GRSF1 loss in THP-1 macrophages promotes senescence-associated transcription in neighboring fibroblasts
Source: Sci Rep. 2025 Aug 14;15:29851. doi: 10.1038/s41598-025-11385-0 (PMC12354690; doi:10.1038/s41598-025-11385-0)
Supplement: Supplementary file 1 — Supplementary Material 1 [file 41598_2025_11385_MOESM1_ESM.pdf]

GRSF1 loss in THP-1 macrophages promotes senescence-associated transcription in neighboring fibroblasts.

Younggi Lee[1a], Seokwoo Jo[2a], Mi-Hee Lim[1a], Sangik Hwang[1], Sohyeon Jang[1], Kyuseok Kim[3], Sung-Jin Yoon[4], Jian Sima[5], M Laura Idda[6], Kyoung Mi Kim[7], Chungoo Park[2#], Myriam Gorospe[8], and Ji Heon Noh[1#]

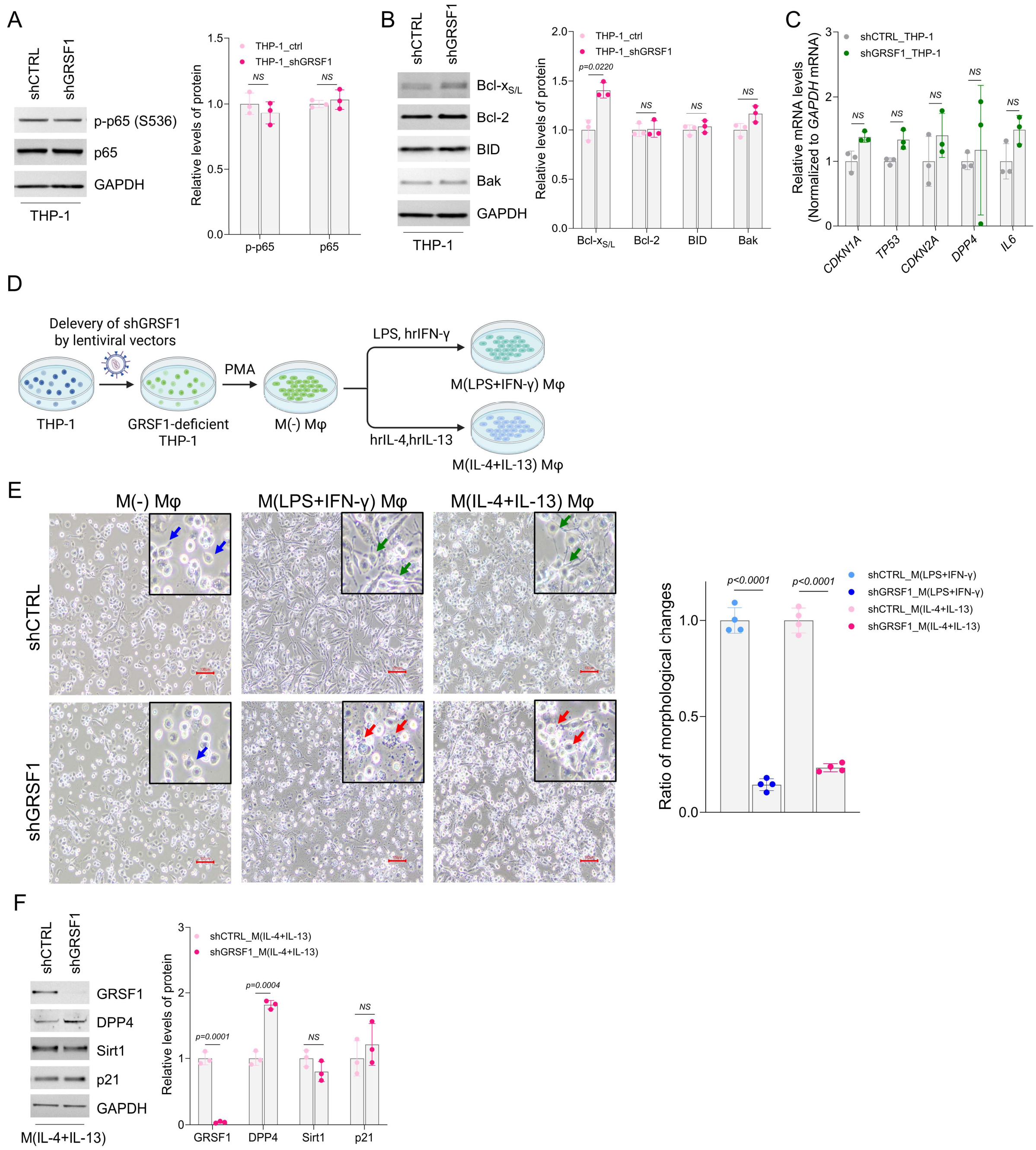

Figure S1. Depleting GRSF1 interferes with macrophage polarization. (A, B) Western blot analyses of (A) phospho-p65 and total p65, and (B) Bcl-2 family proteins (Bcl-xS/L, BCL-2, BID, Bak) in GRSF1-deficient and control THP-1 monocytes (left panel). GAPDH served as a loading control. Quantitative data are shown as bar graphs (right panel). (C) RT-qPCR analysis showing steady-state levels of *CDKN1A, TP53, CDKN2A, DPP4,* and *IL6* mRNAs in control and GRSF1-deficient THP-1 monocytes. (D) Schematic representation of THP-1 monocytes differentiation protocol into M(-), M(LPS+IFN-γ), and M(IL-4+IL-13) macrophages. (E) Morphological characteristics of M(-), M(LPS+IFN-γ) and M(IL-4+IL-13) macrophages were observed via light microscopy. Scale bar: 100 μm. (F) Western blot analysis of GRSF1 and senescence-associated proteins (DPP4, Sirt1, p21) in control and GRSF1-deficient M(IL-4+IL-13) macrophages (left panel). GAPDH was used as a loading

control. Band intensities were quantified and presented as bar graphs (right panel). Data in (A-D) represent the means ± SD from three independent experiments.

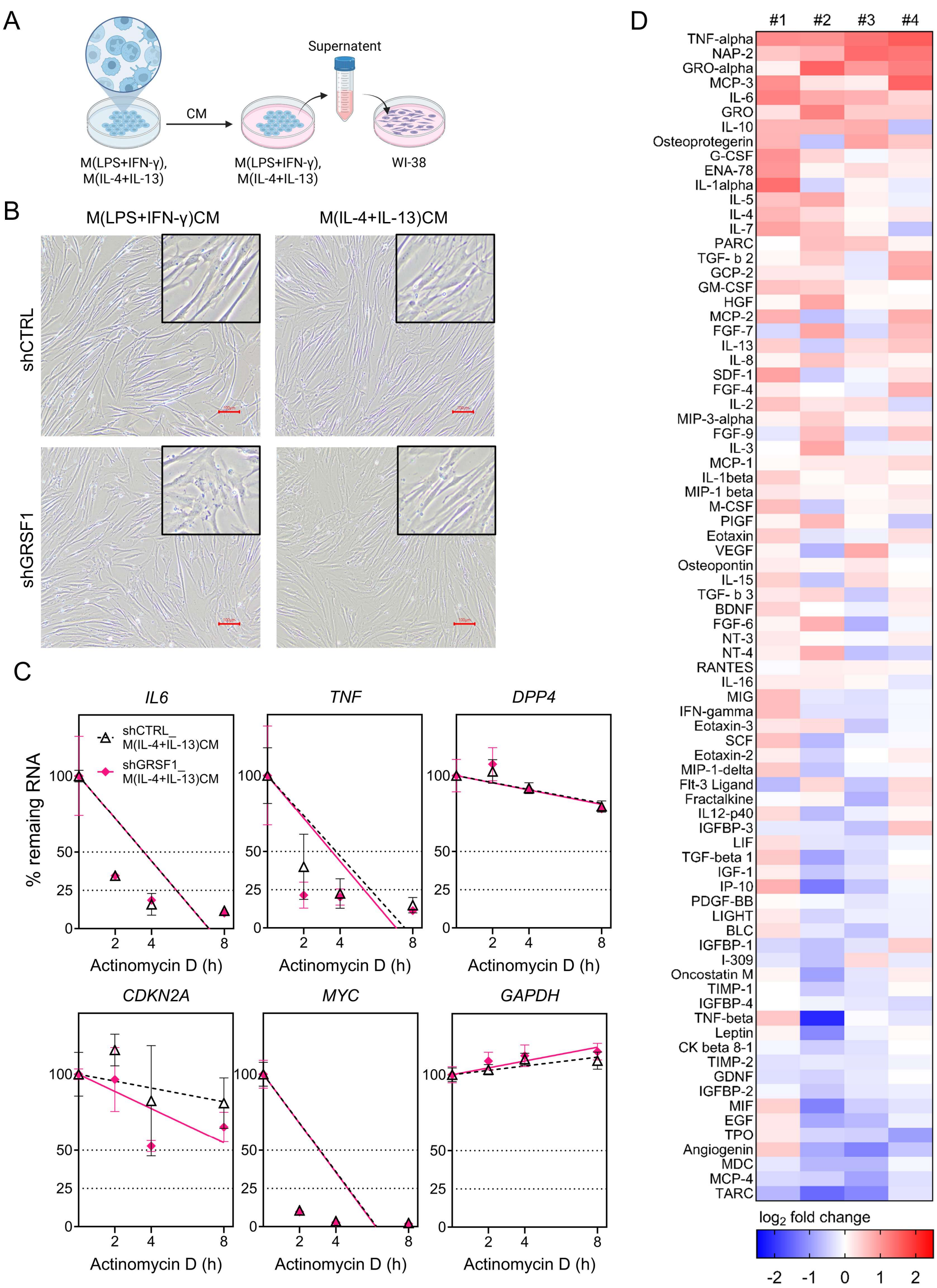

Figure S2. GRSF1-deficient macrophages induce the senescence in neighboring fibroblasts. (A) Experimental workflow illustrating preparation of conditioned media (CM) macrophages (M(LPS+IFN-γ), M(IL-4+IL-13)) derived from control or GRSF1-deficient THP-1 monocytes, and subsequent treatment of WI-38 fibroblasts. (B) Representative morphology of WI-38 fibroblasts after 24 h incubation with indicated CM, observed by light microscopy. Scale bar: 100 µm. (C) Stability of *IL6, TNF, DPP4, and CDKN2A* mRNAs in WI-38 fibroblasts incubated with CM, assessed by RT-qPCR following actinomycin D (2 µg/mL) treatment for indicated times. *MYC* and *GAPDH* mRNAs served as controls for

unstable and stable transcripts, respectively. (D) Heat map showing Log_2 fold-changes of cytokines in CM from GRSF1-deficient versus control M(IL-4+IL-13) macrophages (n = 4). Data in (C) represent the means ± SD from three independent experiments.

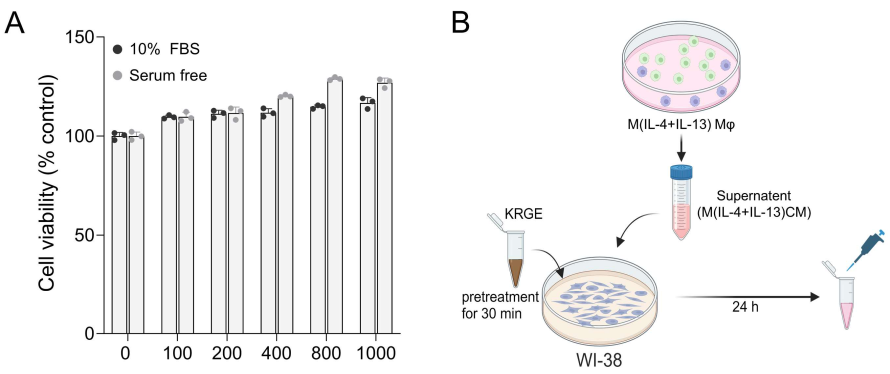

Figure S3. The altered inflammatory environment modulates NF-κB-dependent transcription. (A) Viability of WI-38 fibroblasts treated with red ginseng extract (RGE; 0-1000 μg/mL) in complete (10% FBS) or serum-free media for 24 hours, assessed by MTS assay. (B) Schematic illustration showing experimental procedure: WI-38 fibroblasts were pretreated with RGE for 30 min, followed by exposure to M(IL-4+IL-13) CM for 24 h. Data in (A) represent means ± SD from three independent experiments.

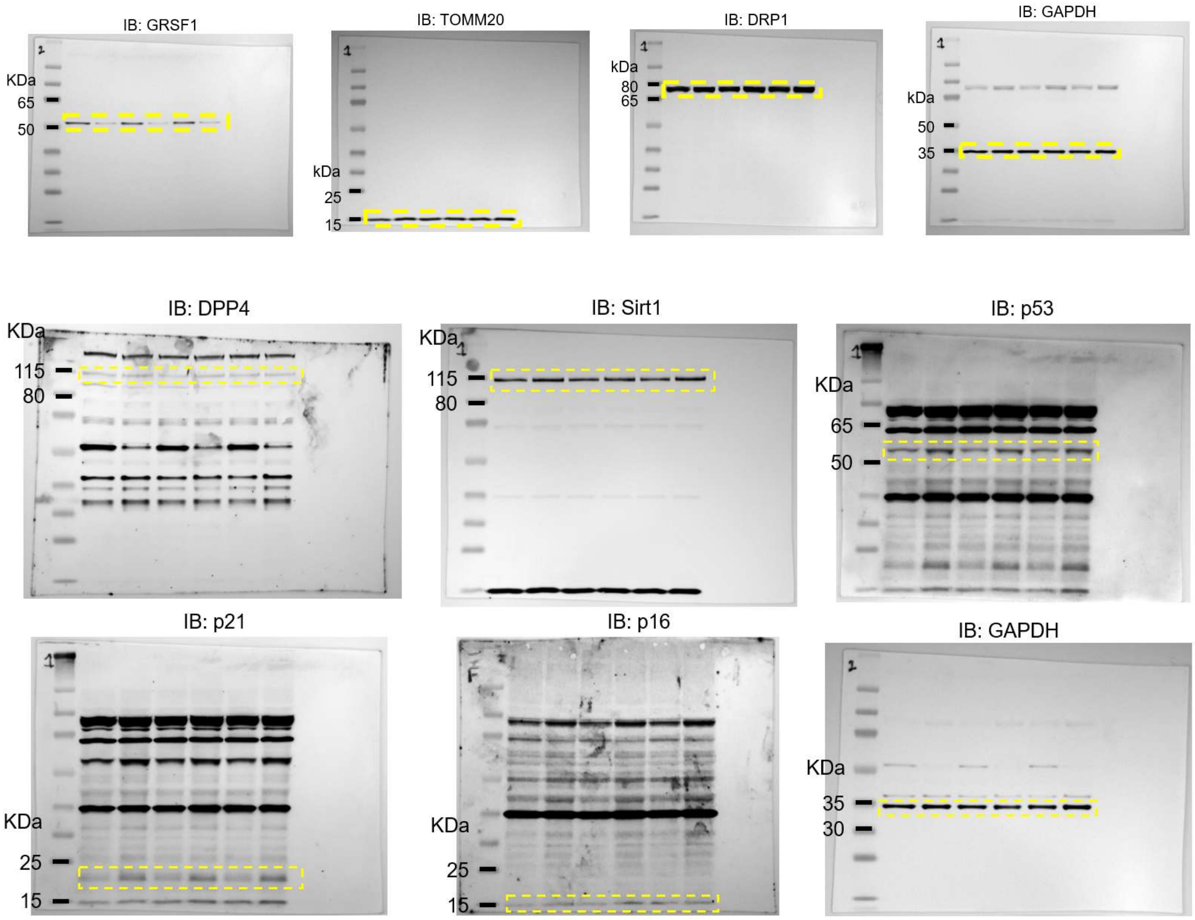

Figure S4. Original Western blot images for Fig 1A and 1B. Dashed rectangles indicate cropped regions presented in Fig 1A and 1B.

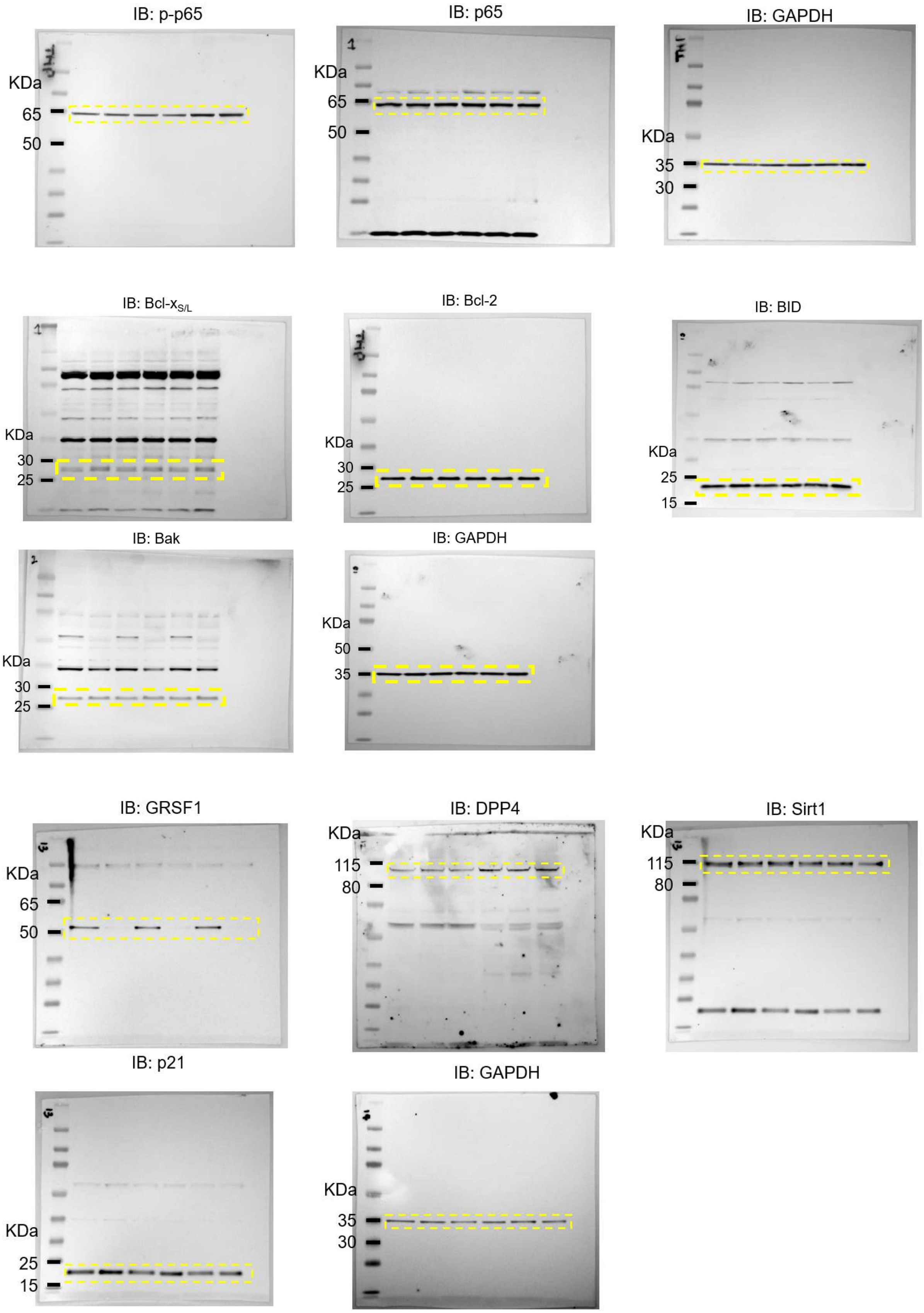

Figure S5. Original Western blot images for Fig S1A, S1B, and S1F. Dashed rectangles indicate cropped regions presented in Fig S1A, S1B, and S1F.

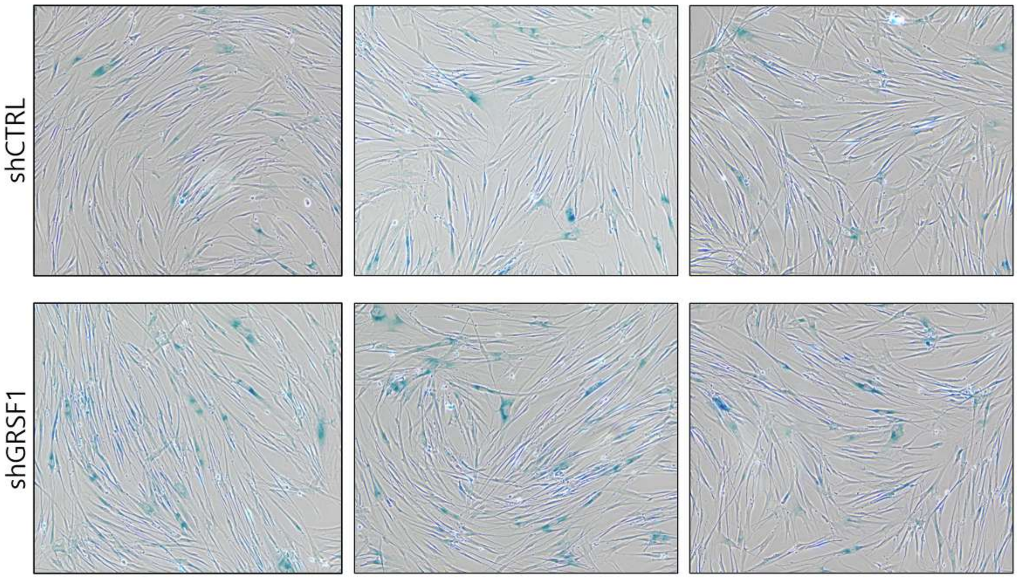

Figure S6. Senescence-associated β-galactosidase (SA-β-gal) staining corresponding to Fig 2B.

Representative images of SA-β-gal-stained WI-38 cells treated with CM from shCTRL_M(IL-4+IL-13) (top) or shGRSF1_M(IL-4+IL-13) macrophages (bottom).

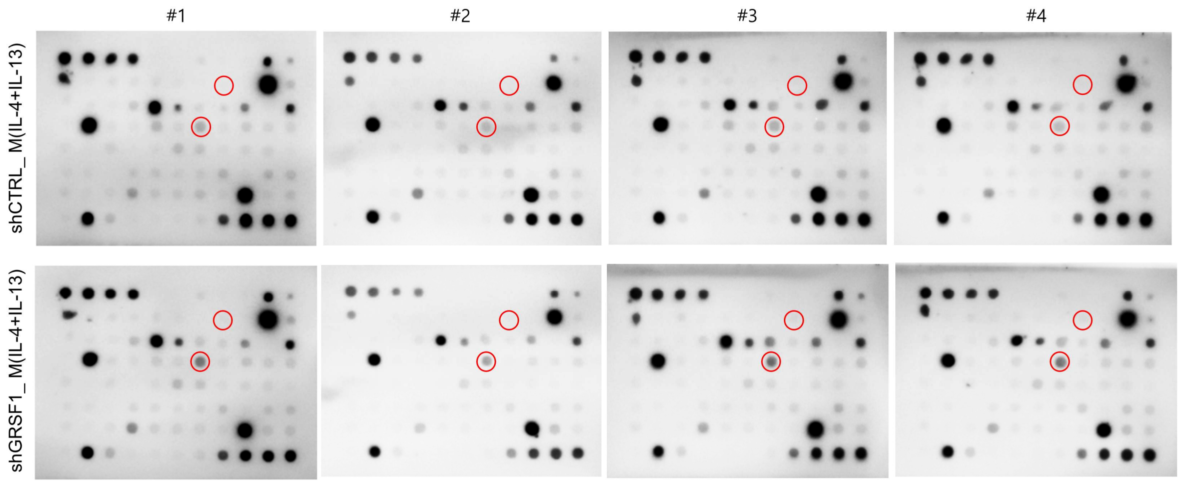

	A	B	C	D	E	F	G	H	I	J	K
1	POS	POS	POS	POS	NEG	NEG	ENA-78 (CXCL5)	G-CSF	GM-CSF	GRO a/b/g	GRO alpha (CXCL1)
2	I-309 (CCL1)	IL-1 alpha (IL-1 F1)	IL-1 beta (IL-1 F2)	IL-2	IL-3	IL-4	IL-5	IL-6	IL-7	IL-8 (CXCL8)	IL-10
3	IL-12 p40/p70	IL-13	IL-15	IFN-gamma	MCP-1 (CCL2)	MCP-2 (CCL8)	MCP-3 (CCL7)	M-CSF	MDC (CCL22)	MIG (CXCL9)	MIP-1 beta (CCL4)
4	MIP-1 delta	RANTES (CCL5)	SCF	SDF-1 alpha	TARC (CCL17)	TGF beta 1	TNF alpha	TNF beta (TNFSF1B)	EGF	IGF-1	Angiogenin
5	OSM	TPO	VEGF-A	PDGF-BB	Leptin	BDNF	BLC (CXCL13)	Ck beta 8-1 (CCL23)	Eotaxin-1 (CCL11)	Eotaxin-2 (CCL24)	Eotaxin-3 (CCL26)
6	FGF-4	FGF-6	FGF-7 (KGF)	FGF-9	FLT-3 Ligand	Fractalkine (CX3CL1)	GCP-2 (CXCL6)	GDNF	HGF	IGFBP-1	IGFBP-2
7	IGFBP-3	IGFBP-4	IL-16	IP-10 (CXCL10)	LIF	LIGHT (TNFSF14)	MCP-4 (CCL13)	MIF	MIP-3 alpha	NAP-2 (CXCL7)	NT-3
8	NT-4	OPN (SPP1)	OPG (TNFRSF11	PARC	PLGF	TGF beta 2	TGF beta 3	TIMP-1	TIMP-2	POS	POS

Figure S7. Original antibody array images corresponding to Fig 2C. Representative raw images from cytokine array experiments performed with CM from shCTRL_M(IL-4+IL-13) (top) or shGRSF1_M(IL-4+IL-13) macrophages (bottom) (n=4). Red circles within each blot highlight IL-6 and TNF-α signals. The table below the images indicates the location of cytokines on the antibody array.

Supplementary Table S1. RT-qPCR Primer sequence

Targer mRNA	Forward primer (5'-3')	Reverse primer (5'-3')
ANKRD1	AGTAGAGGAACTGGTCAC	TGGGCTAGAAGTGTCTTCAGAT
ANRIL	CTCAGACCAGTGACTTAGATTG	GATCCAGTGGTGGATGTTG
CD204	GGAGATGAGGAGGAATACCCAG	CCCACTGCTCCATACTTGGTT
CDKN1A	CCTGCCCAAGCTCTACCTT	AAGGCAGAAGATGTAGAGC
CDKN2A	CGGTCGGAGGCCGATCCAG	GCGCCGTGGAGCAGCAGCAGCT
DPP4	CAAATTGAAGCAGCCAGACA	CACACTTGAACACGCCACTT
GAPDH	CCAAAATCAAGTGGGGCGAT	GGCAGAGATGATGACCCTTT
IL1B	CTACAGCTGGAGAGTGTAGA	GAACTGGGCAGACTCAAAT
IL23A	AGTGGGACACATGGATCT	TGTTGTCCCTGAGTCCTT
IL6	TACTCGGCAAACCTAGTGCG	GTGTCCCAACATTCATATTGTCATT
IL8	GAGTGGACCACACTGCGCCA	TCCACAACCCTCTGCACCCAGT
IRF4	GCTTGTGAAAATGGTTGCCAG	GCAGACCTTATGCTTGGCTC
MIR31HG	TTCCCAGTTTCAGACCACCCT	ACACTGGCCTTGAGGAGGTAT
MRC1	AGTGATGGGACCCCTGTAAC	CCAGTACCCATCCTTGCCTT
MYC	GACTCTGAGGAGGAACAAGA	TGATCCAGACTCTGACCTTT
NKILA	TGCTTTGGAAGGAGCATAG	GAACTGGGTGTCCTGTATTT
PACER	TTGCAGGGACGCTAAATG	GTATCTCCTATGAAGGGCTAGT
pre-DPP4	AAACTGCGACTCGCTTAC	CAGGACCATTGAGGTTACG
pre-IL6	CACACAGACAGCCACTCACC	GAACCCAGCAAAGACCTCCT
pre-IL8	GGACTTAGACTTTATGCCTGAC	TCCACAACCCTCTGCACCCAGT
pre-TNFA	GTAGCCCATGTTGTAGGTAAG	CAAGTTCTGCCTACCATCAG
TNFA	CTGCCTGCTGCACTTTGGAG	ACATGGGCTACAGGCTTGTCACT
TP53	CCCAAGCAATGGATGATTTGA	GGCATTCTGGGAGCTTCATCT
